# Fitness age outperforms body mass index in differentiating aging patterns and health risk profiles of healthy adults aged 51–80 years

**DOI:** 10.1007/s11357-024-01125-z

**Published:** 2024-03-18

**Authors:** Manca A., Ventura L., Martinez G., Morrone M., Boi A., Fiorito G., Mercante B., Cano A., Catte M. G., Cruciani S., Pozzati C., Uccula A., Ginatempo F., Maioli M., Delitala A. P., Solinas G., Zinellu A., Carru C., Deriu F.

**Affiliations:** 1 Department of Biomedical Sciences, University of Sassari, Viale S. Pietro 43/B, 07100 Sassari, Italy; 2 IRCCS Ospedale Pediatrico Giannina Gaslini, Genoa, Italy; 3 Department of History, Human Sciences and Education, University of Sassari, Sassari, Italy; 4 Department of Medicine, Surgery and Pharmacy, University of Sassari, Sassari, Italy; 5 Unit of Endocrinology, Nutritional and Metabolic Disorders, AOU Sassari, Sassari, Italy

**Keywords:** Biological aging, Fitness age, Risk scores, Comorbidity, Cardiovascular diseases

## Abstract

Physical fitness has been extensively shown to strongly associate with general health status and major health risks. Here we tested the ability of a novel estimate of fitness age (FitAge) to differentiate aging trajectories.

This study aimed at (1) testing the ability of FitAge to differentiate aging patterns among decelerated, normal, and accelerated agers in selected health domains, (2) estimating the risk for developing major health issues depending on the aging trajectory, and (3) comparing FitAge to body mass index (BMI) categorization in differentiating healthy from unhealthy aging patterns.

A total of 176 volunteers participated in this cross-sectional study. Participants underwent clinical screening and a comprehensive assessment of body composition, nutritional and health-related status, cognitive functioning, and haematochemical analyses with routine tests, oxidative stress, and inflammation markers. Scores for major health risks were also computed.

FitAge outperformed BMI in estimating major health risk scores and was able to differentiate decelerated from normal and accelerated agers for health risk profile and several physiological domains. Body composition, immune system activation, and inflammation markers emerged as those variables flagging the largest differences between decelerated and accelerated aging patterns.

The novel estimate of biological aging can accurately differentiate both in women and men decelerated from accelerated agers in almost all the domains scrutinized. Overall, decelerated aging is linked to positively oriented features which associate with reduced risk of developing major health issues.

The present findings have potential relevance and practical implications to identify individuals at higher risk of accelerated aging according to their FitAge estimated via simple and cost-effective motor tests.

**Supplementary Information:**

The online version contains supplementary material available at 10.1007/s11357-024-01125-z.

## Introduction

Converging evidence from epidemiological studies suggests that an active lifestyle is associated with a lower risk of cardiovascular events and mortality [1]. A recent cohort study of 4840 US adults aged 40 to 85 years estimated that approximately 110,000 deaths per year could be prevented if physical activity is increased by a small amount (*i.e*., 10 min per day) [2]. In a population-based cohort study of 14,599 UK healthy participants, it was found that healthy middle-aged and older adults, and also individuals with cardiovascular disease and cancer, could gain substantial longevity benefits by becoming more physically active, irrespective of past physical activity levels and established risk factors [3]. Thus, physical fitness (PF) is considered a major determinant of health status that even surpasses other cardiovascular or sociodemographic determinants classically associated with adverse health outcomes [4]. PF declines with age at different individual rates [5]. It has been shown that those who preserve PF while aging are at lower risk for morbidity and tend to live longer [6] [7]. The relationship between PF and aging quality seems of utmost importance as the level of physical activity is a modifiable factor that can act as a potent modulator of the aging trajectory. Considerable impact on population health can be attained with consistent engagement in physical activity during mid to late life. Consequently, health status can be predicted and monitored by assessing PF [8].

A recent cross-sectional study of Italian healthy adults aged 51–80 years introduced a comprehensive and novel measure of biological aging based on different components of fitness. Specifically, using a linear regression model with elastic net penalization previously employed to define the epigenetic age [9], participants’ biological/fitness age (FitAge) was predicted considering the results of six motor-functional fitness tests (Four Square Step Test, Timed Up and Go, 10-Meter Timed Walk, 6-Minute Walk Test, maximum voluntary isometric contraction of the dominant quadriceps, handgrip test) as predictors [10]. Accordingly, age acceleration (FitAA) was defined as the residuals of the regression of FitAge on chronological age, considered dependent variable. This allowed categorization of the participants into three categories: (1) decelerated agers: individuals with FitAA scores lower than − 2.5 years; (2) normal agers: individuals with FitAA scores ranging from − 2.5 to 2.5 years; and (3) accelerated agers: those with FitAA higher than 2.5 years [10].

Based on the above background, the objective of the present study was to test the ability of the newly introduced categorization of biological aging (FitAge) to differentiate among decelerated, normal, and accelerated agers over a comprehensive set of health domains (body composition, nutritional status, general health, cognitive status, hematochemical parameters). Our starting hypothesis was that decelerated agers would exhibit significantly healthier features and scores than accelerated agers in most of the domains tested. Additionally, drawing on the findings of a recent systematic review (11), which indicated a stronger role for fitness levels than fatness as estimated with body weight and height, we also expected that FitAge would outperform the body mass index (BMI), which is universally employed to estimate body weight and fatness, and used to predict major health risks including cardiovascular diseases, comorbidities, and all-causes mortality [11]. With such hypotheses being confirmed, the novel FitAge estimates might represent a robust index to monitor both PF status and the aging trajectory, employing a small set of non-invasive, low-cost validated motor tests.

## Methods

### Study design, participants selection, and clinical examination

The study design was set as observational cross-sectional. The study had an overall duration of 2 years and took place at the Department of Biomedical Sciences, University of Sassari, Italy from January 2021 to December 2023. The current study was advertised via public engagement events to obtain volunteers. Eligible participants were required to be over 50 years old at the time of the examination (from February 2021 to December 2021) and have no medical, physical, or cognitive condition that would interfere with participation in the functional assessments. After a preliminary telephone interview the first 300 respondents deemed apparently eligible were recalled to undergo clinical examinations. All subjects were evaluated by a geriatric specialist against the eligibility criteria. Respiratory, rheumatologic, neurological, cardiovascular, musculoskeletal, neoplastic, and metabolic conditions were thoroughly investigated and set as exclusion criteria. Additional confounding factors that may influence health outcomes, such as the participant’s smoking history, level of education, and current pharmacological therapy, were also checked and recorded.

One hundred and seventy-six volunteers were deemed eligible and participated in the study. The Institutional Review Board and the Clinical Research Ethics Committee approved all procedures involving human subjects (ID: PG/2020/16846), which were carried out in accordance with the Declaration of Helsinki. Written informed consent was obtained from each participant before inclusion and participation in the tests.

### Participants’ assessments

#### Body composition

Body composition was estimated employing BMI calculations (weight/height^2^ in meters) and quantified by bioelectrical impedance vector analysis (BIVA) (BIA 101, Akern, Italy). The subjects were tested supine, after a 5-min rest, on a non-conducting surface with their arms slightly abducted away from their trunk and the legs separated. Absolute and percent body cell mass (BCM), fat mass (FM), fat free mass (FFM), total body water (TBW), extracellular water (ECW) and intracellular water (ICW), skeletal muscle mass (SMM), and appendicular skeletal muscle mass (ASMM) were estimated.

### Nutritional status

Nutritional status was assessed by the Mini Nutritional Assessment (MNA) inventory and dietary habits evaluation. MNA was administered to evaluate the risk of malnutrition (17–23.5 points), protein-calorie malnutrition (< 17 points), or adequate nutritional status (> 23.5 points) [12]. Dietary habits were assessed through validated questionnaires of adherence to Mediterranean Diet [13, 14] and through the 7-Day Food Diary which captures data immediately after principal meals and snacks throughout the day, overcoming potential memory bias.

### Health and cognitive status

Health-related status was investigated trough a set of self-reported scales. The Italian validated version of the Geriatric Depression Scale (GDS) [15] was used to assess the subject’s depressive symptoms. Quality of life, functional status, and well-being were measured using the Italian short-form health survey (SF-36) [16]. Cognitive status was assessed using the Italian version [17] of the Montreal Cognitive Assessment (MOCA) [18]. The sleep quality was assessed using the Italian version [19] of the Pittsburgh Sleep Quality Index (PSQI) [20].

### Hematochemical tests

Blood samples were drawn by the laboratory analysis of the University Hospital of Sassari. A complete haematochemical analysis was performed to appraise the main blood constituents such as white blood cells (WBC), red blood cells (RBC), hemoglobin (HGB), hematocrit (HCT), eosinophils (EOS), large unstained cells (LUC), urea, creatinine, alkaline phosphatase, serum iron, and C-reactive protein (CPR).

*ELISA assays*—The concentrations of interleukin 6 (IL-6), leptin, tumor necrosis factor (TNF-α), and insulin-like growth factor 1 (IGF-1) were determined using Standard ABTS EDK Human IL-6 Kit (PeproTech EC, Ltd., London, UK), Standard ABTS EDK Human Leptin Kit (PeproTech EC, Ltd., London, UK), Human TNF-α Mini TMB ELISA Development Kit (PeproTech EC, Ltd., London, UK), and Human IGF-I ELISA Kit (Sigma-Aldrich Chemie GmbH, Germany), respectively. One hundred microliters of each sample was incubated in a pre-treated plate for 2 h at room temperature (RT). After three washing steps in Phosphate-Buffered Saline (PBS), detection antibody was added in each well for 2 h at RT and then removed and replaced by Streptavidin–Horseradish Peroxidase (HRP) for 30 min at RT. Antibody was then washed three times and liquid substrate incubated at RT for 20 min. Color development was analyzed at 450 nm using a plate reader (Akribis Scientific, Common Farm, Frog Ln, Knutsford, UK). Standard curves were prepared according to the manufacturer’s instructions. Each sample was assayed in duplicate, and values were expressed as the mean ± standard deviation (SD) of 2 measures per sample.

*Oxidative stress indicators*—Blood samples were collected using tubes containing ethylenediaminetetraacetic acid at room temperature. Immediately after collection, the samples were centrifuged at 1500 × g for 10 min, and the plasma was removed and stored at − 80 °C until tested. Paraoxonase (PON-1) activity was determined by using paraoxon (O,O-diethyl-O-p-nitrophenyl phosphate) as a substrate and measuring the increase in absorbance at 412 nm due to the formation of 4-nitrophenol. Enzyme activity was calculated from the molar extinction coefficient (17,000 M^−1^ cm^−1^) with 1 nmol of 4-nitrophenol formed per minute used as the unit of enzyme activity. Protein-SH (PSH) determination was performed spectrophotometrically at 405 nm using Ellman’s reagent (DTNB, 5,5′-dithiobis-2-nitrobenzoic acid) and a standard curve obtained using standard solutions of reduced glutathione (GSH). PSH levels were normalized against the total amount of plasma proteins measured by Lowry’s method at 750 nm. Malondialdehyde (MDA) and other aldehydes produced by lipid peroxidation induced by hydroxyl free radicals were measured spectrophotometrically by the thiobarbituric acid reactive substances (TBARS) method forming an MDA-TBA2 adduct that absorbs strongly at 532 nm. Plasma homocysteine levels were assessed by ELISA kit (antibodies-online GmbH, Germany), according to the manufacturer’s instructions.

### Health risk scores

To estimate the risk for cardiovascular diseases (CVD) three composite risk scores predictive of 10-year risk were computed: the Framingham Risk Score (FRS) for coronary heart disease [21], the CVD risk from the American College of Cardiology (ACC) and American Heart Association (AHA) [22], and the 10-year risk of atherosclerosis based on the Multi-Ethnic Study of Atherosclerosis (MESA) study [23].

The risk of diabetes type 2 [24], the 10-year mortality risk score [25], and the Charlson Comorbidity Index (CCI) [26] were also calculated.

### Statistical analysis

Data analyses were performed using SPSS 26.0 software (IBM, New York, USA). Power analyses were conducted using G*Power 3.1.9.2 software [27]. Normality of data was analyzed with the Shapiro–Wilk test. Levene’s test was performed to test for homogeneity of variances. General linear model univariate analysis of variance (ANOVA) was used to test differences in the outcomes of interest depending on the FitAge categorization over three levels: decelerated, normal, and accelerated aging [10]. Briefly, individuals were divided into the above categories based on the age acceleration displayed through a coreset of fitness tests (FitAA). As introduced in the background, decelerated agers were those individuals with FitAA scores lower than − 2.5 years, normal agers were individuals with FitAA scores ranging from − 2.5 to 2.5 years, and accelerated agers were those with FitAA higher than 2.5 years [10].

The main effect of Sex and FitAge*Sex interactions was also tested, followed by Bonferroni-adjusted pairwise comparisons. The direct effect of body composition based on body weight and height and the derived BMI categories (≤ 18, underweight; 18.1 to 24, normoweight; 24.1 to 29.9, overweight; ≥ 30, obese) was also tested along with the potential FitAge*BMI and sex interaction. Bonferroni-corrected post hoc tests were conducted to locate the main source of the observed significant difference. The non-parametric Kruskal–Wallis test was employed when the assumptions of normality of the data were violated.Correlation analysis was also run to measure the bivariate association of FitAge and BMI with the calculated scores for major health risks (FRS; ACC; AHA; MESA; diabetes; CCI). Pearson’s or Spearman’s *r* coefficients were computed depending on data distribution.

For all tests, statistical significance was set at the conventional threshold of 0.05.

## Results

### Participants

The analyzed sample included 176 volunteers (72 men and 104 women; mean age 66.48 ± 7.77 years) divided in three groups according to the categorization based on FitAA (*n* = 43 decelerated, *n* = 97 normal, and *n* = 36 accelerated). Table 1 summarizes the study sample characteristics including anthropometric and sociodemographic variables such as polypharmacy, smoking status, and educational level at study entry. Tables 2–5 report the main results of the study according to the domain examined, reporting only data for those variables showing significant differences among groups according to FitAA. Data from all those variables for which no significant differences were observed are presented in the Supplementary Materials 1–3.

**Table 1:** Main anthropometric, clinical, and sociodemographic characteristics of the participants

Variable			FitAA status			
			Total (*n* = 176)	Decelerated (*n* = 43)	Normal (*n* = 97)	Accelerated (*n* = 36)
Sex	Women		*n* = 104	*n* = 19	*n* = 63	*n* = 22
	Men		*n* = 72	*n* = 24	*n* = 34	*n* = 14
Age (*years*)	**Whole sample**		**66.48 ± 7.77**	**68.14 ± 5.62**	**65.84 ± 7.40**	**65.47 ± 10.14**
	Women		66.97 ± 7.46	68.42 ± 5.71	66.48 ± 6.74	66.0 ± 9.79
	Men		65.74 ± 8.2	67.92 ± 5.66	64.65 ± 8.47	64.5 ± 11.13
Fitness age (*years*)	**Whole sample**		**66.33 ± 4.57**	**63.59 ± 2.36**	**66.12 ± 3.70**	**70.40 ± 5.98**
	Women		66.73 ± 4.41	63.56 ± 2.37	66.35 ± 3.48	70.55 ± 5.49
	Men		65.74 ± 4.77	63.62 ± 2.4	65.7 ± 4.10	70.11 ± 7.04
Fitness age acceleration (*years*)	**Whole sample**		**0.01 ± 3.04**	** − 3.54 ± 0.92**	** + 0.01 ± 1.40**	** + 4.44 ± 2.27**
	Women		+ 0.22 ± 2.96	− 3.70 ± 1.03	− 0.05 ± 1.41	+ 4.37 ± 2.0
	Men		− 0.33 ± 3.17	− 3.42 ± 0.83	+ 0.12 ± 1.39	+ 4.59 ± 2.78
Weight (*kg*)	**Whole sample**		**69.44 ± 13.29**	**68.76 ± 13.24**	**68.78 ± 13.74**	**72.40 ± 12.30**
	Women		63.55 ± 10.41	58.32 ± 8.36	63.29 ± 10.16	68.67 ± 11.10
	Men		78.35 ± 12.22	77.02 ± 10.22	78.96 ± 13.81	79.25 ± 11.83
Height (*m*)	**Whole sample**		**1.595 ± 0.011**	**1.623 ± 0.095**	**1.585 ± 0.120**	**1.597 ± 0.010**
	Women		1.530 ± 0.09	1.537 ± 0.046	1.531 ± 0.106	1.535 ± 0.05
	Men		1.692 ± 0.072	1.690 ± 0.064	1.686 ± 0.08	1.711 ± 0.066
Body mass index (*kg/m*^*2*^)	**Whole sample**		**26.97 ± 3.95**	**25.93 ± 3.29**	**26.81 ± 4.11**	**28.36 ± 3.72**
	Women		26.77 ± 4.17	24.7 ± 3.57	26.4 ± 4.06	29.01 ± 3.65
	Men		27.27 ± 3.61	26.9 ± 2.75	27.57 ± 4.15	27.15 ± 3.68
Blood pressure—diastolic (*mmHg*)	**Whole sample**		**82.49 ± 8.83**	**80.93 ± 8.54**	**82.48 ± 8.07**	**84.18 ± 11.08**
	Women		81.34 ± 8.93	78.42 ± 8.0	81.49 ± 8.2	82.86 ± 11.46
	Men		84.23 ± 8.43	82.92 ± 8.59	84.32 ± 7.6	86.58 ± 10.41
Blood pressure—systolic (*mmHg*)	**Whole sample**		**130.76 ± 15.08**	**132.81 ± 13.74**	**130.44 ± 15.72**	**129.26 ± 15.28**
	Women		128.66 ± 15.39	128.47 ± 13.18	128.89 ± 15.68	128.32 ± 17.38
	Men		133.93 ± 14.12	136.25 ± 13.45	133.32 ± 15.61	131.0 ± 10.88
Polypharmacy	**Whole sample**		**1 (0.14)**	**1 (0.26)**	**1 (0.17)**	**1.50 (0.39)**
	Women		2 (0.18)	1 (0.43)	2 (0.22)	2 (0.44)
	Men		1 (0.21)	2 (0.30)	1 (0.24)	1 (0.78)
Smoking status	***Smoking***	**Whole sample**	**14.53% (25)**	**24%**	**52%**	**24%**
		Women	72% (18)	100.0%	76.92%	33.33%
		Men	28% (7)	0.0%	23.8%	66.66%
	***Not smoking***	**Whole sample**	**85.47%**	**25.17%**	**54.42%**	**19.05%**
		Women	57.82%	35.14%	62.5%	71.43%
		Men	42.17%	64.86%	37.5%	28.57%
	***Never smoked***	**Whole sample**	**65.99%**	**19.59%**	**57.73%**	**21.65%**
		Women	58.76%	12.28%	59.65%	26.32%
		Men	41.24%	30.0%	55.0%	15.0%
	***Have smoked***	**Whole sample**	**43.5%**	**32.4%**	**50.0%**	**17.6%**
		Women	56.0%	21.43%	57.14%	17.86%
		Men	44.0%	54.55%	36.36%	9.09%
Educational level	***Primary school***	**Whole sample** Women Men	**5.3%**	**23.53%**	**46.47%**	**24.7%**
			66.66%	75.0%	75.0%	50.0%
			33.33%	25.0%	25.0%	50.0%
	***Middle school***	**Whole sample**	**23.8%**	**17.5%**	**52.5%**	**30.0%**
		Women	70.0%	28.57%	71.43%	91.66%
		Men	30.0%	71.43%	28.57%	8.33%
	***High school***	**Whole sample**	**46.4%**	**21.8%**	**60.3%**	**17.9%**
		Women	55.7%	35.29%	61.7%	57.14%
		Men	44.3%	64.71%	38.3%	42.9%
	***Tertiary* + **	**Whole sample**	**25.0%**	**33.3%**	**52.4**	**14.3%**
		Women	59.5%	57.14%	68.18%	33.33%
		Men	40.48%	42.86%	31.82%	66.66%

All data are presented as mean ± standard deviation, except for *Sex, Polypharmacy, and smoking and educational levels*, shown as count (*n*), median and (standard error), percentage, respectively

*Abbreviations: kg* kilograms,* m* meters, *mmHg* millimeters of mercury

**Table 2:** Correlation matrix of FitAge and BMI with health risk scores

Variables	FRS	ACCAHA	Stern diabetes risk	MESA	CCI	Mortality score
*FitAge*	.444***	.672***	.181	.439***	.504***	.660***
*BMI*	.293***	.337***	.440***	.287***	.117	.224**

*Abbreviations: FRS* Framingham risk score coronary heart disease*, ACCAHA* American College of Cardiology and American Heart Association cardiovascular diseases risk score*, MESA* Multi-Ethnic Study of Atherosclerosis risk score*, CCI* Charlson Comorbidity Index*, FitAge* fitness age*, BMI* body mass index

* Significant for *p*<0.05

** Significant for *p*<0.005

*** Significant for *p*<0.001

Overall, one-way ANOVA (factor: three-level BMI categorization: normoweight, overweight, obese) revealed significant main effects for BMI on few selected variables (WBC_count: *p* = 0.005; LUC%: *p* = 0.04), unlike FitAge for which significant main effects were detected for most of the outcomes tested, as detailed below and in Table 3–5. No BMI*FitAA interactions were observed for any of the outcomes nor in women neither in men.

**Table 3:** Significant group differences in body composition domain according to FitAA status and sex

Variable		FitAA status						Statistics		
		Total (*n* = 176)	Decelerated (*n* = 43)		Normal (*n* = 97)	Accelerated (*n* = 36)		FitAA main effect		Post hoc multiple comparisons
								*F* value	*p* value	
BCM (%)	**Whole sample**	**38.35 ± 5.36**		**41.61 ± 4.95**	**37.71 ± 5.16**		**35.97 ± 4.62**	13.641	< 0.0005	Normal < Decel *p* < 0.0005 Accel < Decel *p* < 0.0005
	Women	35.5 ± 3.96		37.98 ± 4.01	35.37 ± 3.77		33.7 ± 3.48	6.357	0.002	
	Men	42.64 ± 4.23		44.48 ± 3.56	42.03 ± 4.59		4.52 ± 2.92	4.797	0.009	
	FitAA*Sex							0.016	0.98	-
FM (%)	**Whole sample**	**27.13 ± 7.93**		**22.92 ± 7.65**	**27.85 ± 7.4**		**30.46 ± 7.76**	10.403	< 0.0005	Normal < Decel *p* = 0.001 Accel < Decel *p* < 0.0005
	Women	31.02 ± 6.67		27.89 ± 8.08	30.97 ± 6.11		34.39 ± 5.29	6.907	0.001	
	Men	21.25 ± 5.77		19.46 ± 5.25	22.07 ± 6.01		22.61 ± 5.65	1.612	0.20	
	FitAA*Sex							0.949	0.39	-
FFM (%)	**Whole sample**	**72.87 ± 7.93**		**77.08 ± 7.65**	**72.15 ± 7.4**		**69.54 ± 7.76**	10.403	< 0.0005	Normal < Decel *p* = 0.001 Accel < Decel *p* < 0.0005
	Women	68.98 ± 6.67		72.71 ± 8.08	69.03 ± 6.11		65.61 ± 5.29	6.907	0.001	
	Men	78.75 ± 5.77		80.54 ± 5.25	77.93 ± 6.01		77.39 ± 5.65	1.612	0.203	
	FitAA*Sex							0.949	0.389	-
TBW (%)	**Whole sample**	**53.47 ± 5.86**		**56.49 ± 5.73**	**52.93 ± 5.40**		**51.09 ± 5.93**	9.709	< 0.0005	Normal < Decel *p* = 0.002 Accel < Decel *p* < 0.0005
	Women	50.49 ± 4.84		53.13 ± 6.00	50.54 ± 4.40		48.08 ± 3.82	6.610	0.002	
	Men	57.94 ± 4.19		59.15 ± 3.9	57.36 ± 4.17		57.13 ± 4.66	1.357	0.260	
	FitAA*Sex							1.060	0.35	
ECW (%)	**Whole sample**	**46.84 ± 3.02**		**45.55 ± 2.29**	**47.15 ± 3.17**		**47.6 ± 3.01**	5.745	0.004	Normal < Decel *p* = 0.010 Accel < Decel *p* = 0.009
	Women	47.84 ± 2.55		47.04 ± 1.73	48.05 ± 2.71		47.95 ± 2.63	1.012	0.366	
	Men	45.33 ± 3.07		44.37 ± 1.98	45.49 ± 3.32		46.89 ± 3.68	3.327	0.038	
	FitAA*Sex							0.951	0.39	-
ICW (%)	**Whole sample**	**53.16 ± 3.02**		**54.45 ± 2.29**	**52.85 ± 3.17**		**52.40 ± 3.01**	5.745	0.004	Normal < Decel *p* = 0.010 Accel < Decel *p* = 0.009
	Women	52.16 ± 2.55		52.96 ± 1.73	51.95 ± 2.71		52.05 ± 2.63	1.012	0.366	
	Men	54.67 ± .3.07		55.63 ± 1.98	54.51 ± 3.32		53.11 ± 3.68	3.327	0.038	
	FitAA*Sex							0.951	0.389	
ECW/ICW ratio	**Whole sample**	**0.89 ± 0.11**		**0.84 ± 0.78**	**0.9 ± 0.12**		**0.91 ± 0.11**	5.852	0.003	Normal < Decel *p* = 0.009 Accel < Decel *p* = 0.009
	Women	0.92 ± 0.1		0.80 ± 0.65	0.93 ± 0.10		0.93 ± 0.1	1.171	0.313	
	Men	0.84 ± 0.11		0.84 ± 0.08	0.84 ± 0.12		0.89 ± 0.14	3.276	0.04	
	FitAA*Sex							0.890	0.412	
ICW/FFM ratio	**Whole sample**	**0.74 ± 0.08**		**0.71 ± 0.07**	**0.74 ± 0.08**		**0.76 ± 0.09**	3.946	0.021	Accel < Decel *p* = 0.018
	Women	0.76 ± 0.08		0.74 ± 0.08	0.76 ± 0.08		0.8 ± 0.07	4.017	0.2	
	Men	0.7 ± 0.06		0.69 ± 0.05	0.70 ± 0.06		0.69 ± 0.08	0.183	0.833	
	FitAA*Sex							1.870	0.16	
SMM (%)	**Whole sample**	**32.36 ± 6.41**		**35.25 ± 6.58**	**31.72 ± 6.03**		**30.51 ± 6.23**	6.639	0.002	Normal < Decel *p* = 0.007 Accel < Decel *p* = 0.004
	Women	28.27 ± 3.83		29.82 ± 5.18	28.28 ± 3.41		26.88 ± 3.25	2.876	0.059	
	Men	38.54 ± 4.13		39.54 ± 3.8	38.1 ± 4.39		37.75 ± 3.95	1.233	0.294	
	FitAA*Sex							0.229	0.8	
ASMM (%)	**Whole sample**	**26.53 ± 3.29**		**28.01 ± 3.10**	**26.2 ± 3.2**		**25.59 ± 3.29**	6.606	0.002	Normal < Decel *p* = 0.007 Accel < Decel *p* = 0.004
	Women	24.53 ± 2.04		25.59 ± 2.26	24.51 ± 1.97		23.69 ± 1.7	3.925	0.022	
	Men	29.54 ± 2.41		29.93 ± 2.20	29.32 ± 2.65		29.38 ± 2.19	0.592	0.554	
	FitAA*Sex							0.845	0.43	

### Associations of FitAge and BMI with health risk scores

The results of bivariate correlations run for FitAge and BMI with health risk scores are summarized in Table 2. FitAge outperformed BMI in all the bivariate correlations, particularly when testing the association with the Mortality score, the only exception being the Stern Diabetes risk for which BMI, unlike FitAge, was significantly associated.

### Comparing decelerated, normal, and accelerated aging trajectories

A significant main effect of Sex was detected for most of the variables tested over the four domains considered. However, no significant Sex*FitAge interactions emerged.

*Body composition*—One-way ANOVA (factor: three-level aging trajectory: decelerated, normal, accelerated) revealed a significant difference between the three groups in all the descriptors of body composition. Post hoc pairwise comparisons with Bonferroni correction for multiple comparisons detected a significant difference favoring the decelerated group over both the normal and accelerated groups in BCM, FM, FFM, TBW, ECW, ICW, ECW to ICW ratio, SMM and ASMM; a significant difference was revealed in ICW to FFM ratio between the decelerated and accelerated groups. All these results are described with statistical details in Table 3.

*Health and cognitive status—*As detailed in Table 4, one-way ANOVA detected significant differences in the Physical functioning, Vitality, and Social Functioning SF-36 sub-scales and in the PSQI. Bonferroni-adjusted pairwise comparisons showed significantly higher functionality scores in decelerated agers as compared to normal and accelerated agers in the Physical Functioning SF-36 sub-scale. For Vitality and Social Functioning SF-36 sub-scales a significant difference emerged between decelerated and normal agers but not between decelerated and accelerated nor normal and accelerated agers. In the PSQI score decelerated agers had significantly higher scores than the accelerated group.

**Table 4:** Significant group differences in health-related outcomes according to FitAA status and sex

Variable		FitAA status				Statistics		
		Total (*n* = 176)	Decelerated (*n* = 43)	Normal (*n* = 97)	Accelerated (*n* = 36)	FitAA main effect		Post hoc multiple comparisons
						***F* value**	***p* value**	
PSQI	**Whole sample**	**8.08 ± 3.48**	**7.35 ± 3.11**	**7.96 ± 3.54**	**9.32 ± 3.51**	3.270	0.04	Decel < Accel *p* = 0.039
	Women	8.93 ± 3.70	8.10 ± 3.63	8.59 ± 3.65	10.63 ± 3.53	3.911	0.022	
	Men	6.78 ± 2.64	6.75 ± 2.54	6.76 ± 3.0	6.92 ± 1.88	0.012	0.988	
	FitAA*Sex					1.288	0.28	
SF-36 Physical Functioning	**Whole sample**	**88.99 ± 11.09**	**94.07 ± 7.43**	**87.60 ± 11.03**	**86.47 ± 13.29**	6.535	0.002	Decel > Normal *p* = 0.004 Decel > Accel *p* = 0.007
	Women	87.36 ± 11.74	95.0 ± 8.16	85.87 ± 11.02	85.0 ± 13.97	6.049	0.003	
	Men	91.45 ± 9.59	93.33 ± 6.70	90.91 ± 10.42	89.17 ± 12.03	0.694	0.501	
	FitAA*Sex					1.460	0.23	
SF-36 Vitality	**Whole sample**	**65.29 ± 16.13**	**70.23 ± 15.88**	**63.13 ± 15.48**	**65.15 ± 17.3**	2.951	0.055	Normal < Decel *p* = 0.049
	Women	63.94 ± 16.03	69.74 ± 12.96	62.30 ± 16.09	63.64 ± 17.74	1.574	0.21	
	Men	67.32 ± 16.17	70.63 ± 18.14	64.7 ± 14.36	67.92 ± 16.85	0.959	0.385	
	FitAA*Sex					0.10	0.90	
SF-36 Social Functioning	**Whole sample**	**80.36 ± 19.59**	**88.08 ± 15.66**	**76.98 ± 21.33**	**80.15 ± 16.32**	4.991	0.008	Normal < Decel *p* = 0.006
	Women	77.67 ± 21.8	86.18 ± 20.37	74.25 ± 22.89	80.11 ± 17.95	3.08	0.049	
	Men	84.42 ± 14.91	88.08 ± 15.66	82.2 ± 17.13	80.21 ± 13.54	1.394	0.251	
	FitAA*Sex					0.549	0.579	

All data are scores, which are shown as mean ± standard deviation

*Abbreviations: FitAA* Fitness Age Acceleration*, PSQI* Pittsburgh Sleep Quality Index*, SF-36* 36-Item Short Form Survey

No other significant differences emerged for GDS, MoCA, and the other SF-36 subscales (Supplementary Material 1).

*Nutritional status—*No significant differences in MNA nor dietary habits emerged among groups (Supplementary Material 2).

*Hematochemical parameters*—Overall, regardless of the Fit_AA group, all participants displayed hematochemical values and scores falling within the physiological ranges. ANOVA showed a significant difference among groups in WBC, HGB, hematocrit, EOS (%), LUC, serum creatinine, cPR, and Il-6. Bonferroni-adjusted pairwise comparisons detected a significant difference between the accelerated and decelerated groups in WBC (lower count in decelerated agers), hematocrit (higher in decelerated agers) and EOS (lower count in decelerated agers), LUC (lower count in decelerated agers), HGB (higher concentration in decelerated agers), serum creatinine (higher concentration in decelerated agers), IL-6 (higher concentration in decelerated agers), and CRP (higher concentration in accelerated agers). All these results and their statistics are detailed in Table 5. No other significant differences were detected in the other hematochemical parameters (Supplementary Material 3).

**Table 5:** Significant group differences in hematochemical parameters according to FitAA status and sex

Variable		FitAA status				Statistics		
		Total (*n* = 176)	Decelerated (*n* = 43)	Normal (*n* = 97)	Accelerated (*n* = 36)	FitAA main effect		Post hoc multiple comparisons
						***F* value**	***p* value**	
WBC (× 10^3/μL)	**Whole sample**	**5.95 ± 1.41**	**5.73 ± 1.34**	**5.86 ± 1.18**	**6.48 ± 1.91**	3.161	0.045	Accel < Decel *p* = 0.021
	Women	5.64 ± 1.19	5.34 ± 1.42	5.63 ± 0.98	5.95 ± 1.48	1.051	0.352	
	Men	6.41 ± 1.58	6.05 ± 1.21	6.3 ± 1.4	7.42 ± 2.27	4.407	0.014	
	FitAA*Sex					1.09	0.339	-
HGB (g/dL)	**Whole sample**	**13.95 ± 1.34**	**14.46 ± 1.27**	**13.76 ± 1.3**	**13.86 ± 1.4**	4.291	0.015	Normal < Decel *p* = 0.013
	Women	13.45 ± 1.11	13.7 ± 0.96	13.45 ± 1.17	13.22 ± 1.03	0.823	0.441	
	Men	14.72 ± 1.29	15.09 ± 1.15	14.35 ± 1.34	14.98 ± 1.28	3.013	0.052	
	FitAA*Sex					1.707	0.18	-
HCT (%)	**Whole sample**	**43.56 ± 4.66**	**45.17 ± 3.31**	**43.28 ± 3.5**	**42.28 ± 7.68**	4.061	0.019	Accel < Decel *p* = 0.022
	Women	41.91 ± 4.81	42.75 ± 2.54	42.24 ± 3.18	40.19 ± 8.68	2.372	0.097	
	Men	46.04 ± 3.09	47.18 ± 2.4	45.27 ± 3.24	45.96 ± 3.41	1.418	0.245	
	FitAA*Sex					1.318	0.27	-
EOS (%)	**Whole sample**	**3.00 ± 1.66**	**2.60 ± 1.12**	**2.99 ± 1.57**	**3.54 ± 2.30**	2.982	0.053	Decel < Accel *p* = 0.047
	Women	2.86 ± 1.67	2.27 ± 1.01	2.92 ± 1.56	3.2 ± 2.29	1.710	0.184	
	Men	3.22 ± 1.64	2.87 ± 1.15	3.14 ± 1.59	4.12 ± 2.3	2.383	0.095	
	FitAA*Sex					0.577	0.56	-
EOS (× 10^3/μL)	**Whole sample**	**0.19 ± 0.14**	**0.16 ± 0.08**	**0.18 ± 0.12**	**0.26 ± 0.21**	5.181	0.007	Decel < Normal *p* = 0.007
	Women	0.17 ± 0.12	0.13 ± 0.07	0.16 ± 0.08	0.22 ± 0.20	2.661	0.073	
	Men	0.23 ± 0.16	0.18 ± 0.08	0.23 ± 0.17	0.32 ± 0.21	3.992	0.02	
	FitAA*Sex					0.217	0.81	-
LUC (× 10^3/μL)	**Whole sample**	**0.14 ± 0.09**	**0.12 ± 0.04**	**0.13 ± 0.05**	**0.18 ± 0.18**	4.552	0.012	Decel < Accel *p* = 0.024 Normal < Accel *p* = 0.018
	Women	0.13 ± 0.1	0.11 ± 0.03	0.12 ± 0.05	0.17 ± 0.2	2.955	0.055	
	Men	0.15 ± 0.08	0.13 ± 0.05	0.14 ± 0.06	0.19 ± 0.14	1.831	0.164	
	FitAA*Sex					0.019	0.98	-
Creatinine (mg/dL)	**Whole sample**	**0.85 ± 0.15**	**0.93 ± 0.13**	**0.82 ± 0.14**	**0.84 ± 0.14**	5.243	0.007	Normal < Decel *p* = 0.006
	Women	0.77 ± 0.07	0.8 ± 0.05	0.77 ± 0.08	0.75 ± 0.06	0.462	0.632	
	Men	0.97 ± 0.14	0.99 ± 0.11	0.94 ± 0.18	0.97 ± 0.13	0.673	0.513	
	FitAA*Sex					0.275	0.76	-
CRP (mg/dL)	**Whole sample**	**0.38 ± 0.16**	**0.37 ± 0.14**	**0.36 ± 0.14**	**0.45 ± 0.20**	3.311	0.039	Normal < Accel *p* = 0.035
	Women	0.39 ± 0.16	0.39 ± 0.16	0.37 ± 0.14	0.44 ± 0.23	2.937	0.056	
	Men	0.37 ± 0.14	0.37 ± 0.14	0.34 ± 0.13	0.45 ± 0.17	0.169	0.845	
	FitAA*Sex					0.672	0.01	-
IL-6 (ng/mL)	**Whole sample**	**733.29 ± 269.86**	**819.16 ± 287.58**	**686.14 ± 238.57**	**755.52 ± 305.60**	3.762	0.025	Normal < Decel *p* = 0.024
	Women	693.66 ± 247.58	707.40 ± 236.53	698.71 ± 250.48	666.784 ± 258.77	0.153	0.858	
	Men	792.44 ± 292.04	911.40 ± 297.77	662.56 ± 216.30	910.81 ± 329.54	7.816	0.001	
	FitAA*Sex					4.891	0.01	-

All data are shown as mean ± standard deviation

*Abbreviations: FitAA* Fitness Age Acceleration*, WBC* white blood cells*, Hgb* hemoglobin*, HCT* hematocrit test*, EOS* eosinophils*, LUC* large unstained cells*, CRP* C-reactive protein*, IL-6* interleukin 6*, μL* microliters*, mg/dL* milligrams/deciliters*, ng/mL* nanograms/milliliters

## Discussion

The main objective of the present study was to compare three groups of healthy adults aged 51–80 years according to their aging trajectory, *i.e.*, decelerated, normal, or accelerated aging over a range of selected health domains. This categorization based on FitAA was introduced in a previous work from the same laboratory where performance in a comprehensive set of motor tests was found to predict biological age in a meaningful and accurate manner [10]. The three groups were contrasted over a range of domains including body composition, hematochemical parameters, cognitive performance, mood, sleep, global health, physical functioning, and nutritional status.

The first finding of this study was that the proposed categorization could differentiate decelerated from accelerated aging, with these trajectories corresponding to significant differences in body composition and hematochemical profile. Indeed, even though all participants were healthy and fell within the physiological range for the variables assessed, older adults with decelerated aging trajectories exhibited significant, positively oriented differences from accelerated agers in almost all the domains scrutinized (e.g., lower cRP; higher HGB, etc.). Conversely, categorization in normoweight, overweight, and obese participants, as estimated by BMI, was not fully capable to differentiate healthy from unhealthy patterns. While BMI scores associated with risk scores for developing major health conditions including type-2 diabetes, and cardiovascular diseases, this estimate was outperformed by the newly introduced categorization based on FitAge. Importantly, FitAge had similar capabilities of differentiating decelerated from accelerated agers in women and men, as suggested by the absence of significant interactions with sex and in spite of the observed differences in most of the domains here evaluated.

### Health-related domain

In our cohort the quality of sleep assessed by PSQI proved poor, with scores exceeding the cutoff of 7 points in all groups, which indicates disturbed sleep [28]. However, significantly lower scores were detected for decelerated compared to accelerated agers, in line with the established knowledge that being physically active improves sleep quality in middle-aged and older adults [29–31]. While decelerated and normal agers displayed a significantly lower BMI (25.9 and 26.3, respectively) than accelerated agers (28.4), all groups fell within the overweight range. Some potential underlying mechanisms that link poor sleep to overweight/obesity and vice versa have been previously described. Poor quality and quantity of sleep lead to altered metabolic and endocrine function [32], for instance in the stress response system as well as in the secretion of hormones such as ghrelin and leptin, which play important roles in appetite regulation and influence food choice and caloric intake [33]. In our study, however, ghrelin was not measured, and leptin levels were comparable among groups, and not associated with poor sleep patterns. It has also been proposed that insufficient sleep, sleep disturbances, and the consequent daytime sleepiness and fatigue may lead to reduced physical activity [33]. Overall, these influences can lead to an imbalance between energy intake and expenditure potentially promoting weight gain.

Regarding self-reported quality of life assessed by the SF-36 questionnaire, decelerated agers displayed significantly higher scores, i.e., higher quality of life, of physical and social functioning. When checking the observed scores against the largest normative dataset available [34], physical functioning of our total cohort was approximately 28% higher, raising to a 36% difference for decelerated agers. Conversely, for the social functioning sub-score, both normal and accelerated agers were below the normative value, whereas decelerated agers showed a 9% higher score.

### Body composition

It is well-established that aging is associated to changes in body composition including increased FM and decreased lean mass [35, 36]. Participants in the decelerated aging group likely counteracted such process as they displayed significantly less FM and more SMM than accelerated or normal agers. The relationship between body composition and frailty has been mainly related to higher levels of inflammation [37] and impairment of muscle quality due to fat infiltration [38]. These two factors have been demonstrated to interact, jointly contributing to the development of insulin and catecholamine resistance, eventually reducing metabolic flexibility, *i.e*., the cellular capacity to switch freely between oxidative substrates in response to nutritional and physiological cues [37, 39]. Our data also revealed that decelerated agers displayed more favorable cellular water compartmentalization, as shown by the significantly lower ECW, higher ICW, and, consequently, lower ECW/ICW ratio. ICW reflects muscle cell mass, whereas ECW represents the sum of interstitial fluid and blood plasma in the extracellular space [40]. An increase in the ECW/ICW ratio, as detected in our normal and accelerated agers, indicates a relative increase in non-contractile tissue, which has been linked to reduced muscle strength and functional capacity, eventually leading to dynapenia and sarcopenia [41, 42]. This ratio, which is now recognized as a valid predictor of global health and functional status, being the lower the better [41, 43], stood out in our cohort as a highly sensitive parameter for distinguishing decelerated from accelerated agers, and decelerated from normal agers, as well.

### Hematochemical domain

While all participants displayed values and scores falling within the physiological range, significant differences emerged for several hematochemical parameters. Decelerated agers displayed significantly higher HGB content and hematocrit. Such findings were expected since these are well-known adaptations for increasing oxygen-carrying capacity that are associated to a physically active lifestyle [44]. The possible underlying mechanisms are proposed to come mainly from bone marrow, including stimulated erythropoiesis with hyperplasia of the hematopoietic bone marrow, improvement of the hematopoietic microenvironment, and hormone- and cytokine-accelerated erythropoiesis induced by cardiovascular exercise training [45].

While WBC count was within the normality intervals (4500–11,000 WBC per microliter) for all three groups, the number of leukocytes was significantly higher among accelerated agers. Even though a 10–13% increase is not suggestive per se of health issues, the results of the Baltimore Longitudinal Study of Aging [46] showed a significant linear increase on the WBC *vs.* mortality curve in all-causes and cardiovascular mortality, starting from WBC count > 3500/μL. In that study the threshold for increased risk was identified for WBC count ≥ 6000/mm^3^. Four categories were thus identified (≤ 3500; 3501–6000; 6001–10,000; ≥ 10,000). Based on this categorization, the three groups of our cohort of healthy aged adults polarized into “low risk” (decelerated agers: 5729 ± 1340; normal agers: 5859 ± 1180) and “moderate risk” (accelerated agers: 6484 ± 1910).

Even though within normality values for all groups (< 5%), eosinophil fraction was found significantly higher in accelerated compared to normal and decelerated agers, suggesting that a subclinical state of low-grade chronic inflammation was present. The same pattern applied to CRP levels, which were found elevated in accelerated agers by 19–23% compared to decelerated and normal groups, respectively. CRP participates in the systemic response to inflammation, and its levels are known to be proportional to cardiovascular and all-causes mortality risks [47]. Also, LUC count was found to differ according to the aging trajectory, with decelerated agers showing a lower count than normal and accelerated agers. LUC are large-sized peroxidase-negative blood cells that tend to rise in number when markers of inflammation in the body increase, i.e., when lymphocytes or monocytes are activated [48]. The same lymphocytes, when activated, are enlarged in size, and can be considered LUC. Overall, the findings of lower LUC counts and other inflammatory markers in decelerated agers are in line with the results of a large report of 51,623 participants from the US National Health and Nutrition Examination Survey, which showed that the rise of inflammatory scores contributes to accelerated aging and multi-morbidity [49].

Decelerated agers presented significantly higher mean serum creatinine. Creatinine is an endogenous substance generated from the nonenzymatic conversion of creatine and creatine phosphate, 95% of which is found in muscles [50]. While serum creatinine is commonly used to estimate glomerular filtration rate and monitor kidney functioning, elevated levels are also found in healthy individuals with increased muscle mass or high meat consumption [51]. Due to the correlation between its levels and muscle mass, serum creatinine has long been used as a surrogate of muscle mass measurements [52, 53]. Creatinine generation is low among individuals who have reduced muscle mass, either constitutionally or disease related. Hence, low creatinine level could be regarded as a proxy of muscle mass and protein-energy wasting, which is a relevant issue in older adults due to the increasing prevalence of sarcopenia and dynapenia beginning from the fifth decade of life.

IL-6 was found significantly higher in decelerated agers. While IL-6 has context-dependent pro- and anti-inflammatory properties [54], the view in the context of exercise is that it has primarily anti-inflammatory effects [55]. IL-6 directly inhibits the expression of TNF-α and IL-1β and is a potent inducer of the interleukin-1 receptor antagonist, which exerts anti-inflammatory activity by blocking IL-1 receptors and thereby preventing signal-transduction of the pro-inflammatory IL-1 [55]. Unlike IL-1β and TNF-α, IL-6 does not upregulate major inflammatory mediators such as nitric oxide or matrix metalloproteinases. Rather, IL-6 appears to be the primary inducer of the hepatocyte-derived acute-phase proteins, many of which have anti-inflammatory properties. Skeletal muscles appear to be a key organ in IL-6 production. Research has demonstrated that IL-6 is produced by contracting skeletal muscles [56] and is released to the circulation in large quantities, being subjected to a very high turnover during muscular exercise [57].

### Aging trajectories and health risk scores

While also BMI was significantly associated with major health risk scores, FitAge emerged as a stronger predictor for most of the calculated risks (5 out of 6), in line with the now widely accepted notion that higher fitness is associated with better health outcomes [8]. Substantial evidence during the last two decades indicates that PF status markedly alters the relationship between body fatness and subsequent major health outcomes [58]. Although clearly excess body weight and low PF are synergistically associated with worse CVD risk factors and increased prevalence of CVD, compared to normal weight-fit individuals, unfit individuals have a twofold higher risk of mortality regardless of BMI. Overweight-fit and obese-fit individuals have a similar mortality risk as normal weight individuals [11], suggesting that researchers, clinicians, and public health officials, while not discarding BMI, should devote more attention to physical activity and fitness-based interventions rather than narrowing strategies to weight-loss driven approaches to reduce CVD and mortality risk.

### Study limitations

This work is not free of limitations. First, cross-sectional designs like the one here employed do not allow to draw causal inferences as they are devised to test potential associations rather than conclusively establishing cause-and-effect relationships. Future studies with a longitudinal observation will have to confirm whether the observed associations hold a clinical meaning. Second, we did not set the study focus only on older adults as also middle-aged adults transitioning into senescence were included. Third, data from a sample of 176 Italian participants from one single center may limit the generalizability and external validity of our findings, thus warranting additional research to explore whether the study findings hold true across a broader population spectrum in terms of more diverse age groups and demographics. Additionally, our aging trajectories were determined by relying mainly on the fitness levels displayed by the participants. Further studies should improve the accuracy and ecological validity of our novel biomarker—the FitAA—by including age accelerators derived from other relevant domains, such as cognitive, metabolic, and hematochemical ones, as well as by validating it against established descriptors of physical fitness such as maximal oxygen uptake and muscle strength. Finally, while a strength of the present study resides in the profiling of cellular senescence for muscle mass and other cells in the body based on fitness status, genetic and molecular analyses could more adequately allow to validate FitAA as well as test experimentally the mechanisms underpinning the multi-level aging process.

## Conclusions

The newly introduced categorization based on FitAge acceleration of biological aging was able to differentiate decelerated from normal and accelerated agers in terms of health risk profile and several physiological domains. In particular, body composition and hematochemical parameters describing immune system activation and inflammation markers emerged as those variables flagging the largest differences between decelerated and accelerated aging trajectories. FitAA, which is a resource efficient index based on simple and cost-effective motor tests that combines different types of performance, all functionally relevant to the aging person (walking ability, dynamic balance, executive functions, muscle strength, and cardiorespiratory fitness, may represent a promising tool for monitoring agers over time, potentially allowing early detection of even subtle motor-functional deteriorations. Prospectively, if interventions are administered to manage age-related functional decline, FitAA could also serve as a relevant endpoint of treatment to evaluate the effectiveness of the proposed therapeutic approaches.

## Supplementary Information

Below is the link to the electronic supplementary material.

Supplementary file1 (DOCX 67 KB)

## Data Availability

All data are stored at the Department of Biomedical Sciences and will be made available by the corresponding author upon reasonable request.

## References

[1] Benjamin EJ, Muntner P, Alonso A, Bittencourt MS, Callaway CW, Carson AP, et al. American Heart Association Council on Epidemiology and Prevention Statistics Committee and Stroke Statistics Subcommittee (2019) Heart Disease and Stroke Statistics—2019 update: a report from the American Heart Association. Circulation 139:e56-e528 10.1161/CIR.0000000000000659 30700139

[2] Saint-Maurice PF, Graubard BI, Troiano RP, Berrigan D, Galuska DA, Fulton JE, Matthews CE. Estimated number of deaths prevented through increased physical activity among US adults. JAMA Intern Med. 2022;182:349–52. 35072698 10.1001/jamainternmed.2021.7755 PMC8787676

[3] Mok A, Khaw KT, Luben R, Wareham N, Brage S. Physical activity trajectories and mortality: population based cohort study. BMJ. 2019;365:l2323. 31243014 10.1136/bmj.l2323 PMC6592407

[4] Sanchez-Sanchez JL, Izquierdo M, Carnicero-Carreño JA, García-García FJ, Rodríguez-Mañas L. Physical activity trajectories, mortality, hospitalization, and disability in the Toledo Study of Healthy Aging. J Cachexia Sarcopenia Muscle. 2020;11:1007–17. 32163233 10.1002/jcsm.12566 PMC7432572

[5] van Oostrom SH, Engelfriet PM, Verschuren WMM, Schipper M, Wouters IM, Boezen M, Smit HA, Kerstjens HAM, Picavet HSJ. Aging-related trajectories of lung function in the general population—The Doetinchem Cohort Study. PLoS ONE. 2018;13:e0197250. 29768509 10.1371/journal.pone.0197250 PMC5955530

[6] Jackson AS, Sui X, Hébert JR, Church TS, Blair SN. Role of lifestyle and aging on the longitudinal change in cardiorespiratory fitness. Arch Intern Med. 2009;169:1781–7. 19858436 10.1001/archinternmed.2009.312 PMC3379873

[7] Ching SM, Chia YC, Lentjes MAH, Luben R, Wareham N, Khaw KT. FEV1 and total cardiovascular mortality and morbidity over an 18 years follow-up population-based prospective EPIC-NORFOLK study. BMC Public Health. 2019;19:501. 31053065 10.1186/s12889-019-6818-x PMC6500069

[8] Myers J, McAuley P, Lavie CJ, Despres JP, Arena R, Kokkinos P. Physical activity and cardiorespiratory fitness as major markers of cardiovascular risk: their independent and interwoven importance to health status. Prog Cardiovasc Dis. 2015;57:306–14. 25269064 10.1016/j.pcad.2014.09.011

[9] Horvath S. DNA methylation age of human tissues and cell types. Genome Biol. 2013;14:R115. 24138928 10.1186/gb-2013-14-10-r115 PMC4015143

[10] Manca A, Fiorito G, Morrone M, Boi A, Mercante B, Martinez G, Ventura L, Delitala AP, Cano A, Catte MG, Solinas G, Melis F, Ginatempo F, Deriu F. A novel estimate of biological aging by multiple fitness tests is associated with risk scores for age-related diseases. Front Physiol. 2023;14:1164943. 37228822 10.3389/fphys.2023.1164943 PMC10203437

[11] Barry VW, Baruth M, Beets MW, Durstine JL, Liu J, Blair SN. Fitness vs. fatness on all-cause mortality: a meta-analysis. Prog Cardiovasc Dis. 2014;56:382–90. 24438729 10.1016/j.pcad.2013.09.002

[12] Vellas B, Villars H, Abellan G, Soto ME, Rolland Y, Guigoz Y, Morley JE, Chumlea W, Salva A, Rubenstein LZ, Garry P. Overview of the MNA—its history and challenges. J Nutr Health Aging. 2006;10:456–63. 17183418

[13] Trichopoulou A, Costacou T, Bamia C, Trichopoulos D. Adherence to a Mediterranean diet and survival in a Greek population. N Engl J Med. 2003;348:2599–608. 12826634 10.1056/NEJMoa025039

[14] Monteagudo C, Mariscal-Arcas M, Rivas A, Lorenzo-Tovar ML, Tur JA, Olea-Serrano F. Proposal of a Mediterranean diet serving score. PLoS ONE. 2015;10:e0128594. 26035442 10.1371/journal.pone.0128594 PMC4452755

[15] Chiesi F, Primi C, Pigliautile M, Baroni M, Ercolani S, Paolacci L, Boccardi V, Mecocci P. Does the 15-item Geriatric Depression Scale function differently in old people with different levels of cognitive functioning? J Affect Disord. 2018;227:471–6. 29156360 10.1016/j.jad.2017.11.045

[16] Apolone G, Mosconi P. The Italian SF-36 health survey: translation, validation and norming. J Clin Epidemiol. 1998;51(11):1025–36. 9817120 10.1016/s0895-4356(98)00094-8

[17] Santangelo G, Siciliano M, Pedone R, Vitale C, Falco F, Bisogno R, Siano P, Barone P, Grossi D, Santangelo F, Trojano L. Normative data for the Montreal Cognitive Assessment in an Italian population sample. Neurol Sci. 2015;36:585–91. 25380622 10.1007/s10072-014-1995-y

[18] Nasreddine ZS, Phillips NA, Bédirian V, Charbonneau S, Whitehead V, Collin I, Cummings JL, Chertkow H. The Montreal Cognitive Assessment, MoCA: a brief screening tool for mild cognitive impairment. J Am Geriatr Soc. 2005;53:695–9. 15817019 10.1111/j.1532-5415.2005.53221.x

[19] Curcio G, Tempesta D, Scarlata S, et al. Validity of the Italian Version of the Pittsburgh Sleep Quality Index (PSQI). Neurol Sci. 2013;34:511–9. 22526760 10.1007/s10072-012-1085-y

[20] Buysse DJ, Reynolds CF 3rd, Monk TH, Berman SR, Kupfer DJ. The Pittsburgh Sleep Quality Index: a new instrument for psychiatric practice and research. Psychiatry Res. 1989;28:193–213. 2748771 10.1016/0165-1781(89)90047-4

[21] D’Agostino RB, Vasan RS, Pencina MJ, Wolf PA, Cobain M, Massaro JM, et al. General cardiovascular risk profile for use in primary care: the Framingham heart study. Circulation. 2008;117:743–53. 18212285 10.1161/CIRCULATIONAHA.107.699579

[22] Goff DC Jr, Lloyd-Jones DM, Bennett G, Coady S, D’Agostino RB, Gibbons R, et al. American College of Cardiology/American Heart Association Task Force on Practice Guidelines. 2013 ACC/AHA guideline on the assessment of cardiovascular risk: a report of the American College of Cardiology/American Heart Association Task Force on Practice Guidelines. Circulation. 2014;129:S49-73. 24222018 10.1161/01.cir.0000437741.48606.98

[23] Budoff MJ, Young R, Burke G, Jeffrey Carr J, Detrano RC, Folsom AR, Kronmal R, Lima JAC, Liu KJ, McClelland RL, Michos E, Post WS, Shea S, Watson KE, Wong ND. Ten-year association of coronary artery calcium with atherosclerotic cardiovascular disease (ASCVD) events: the multi-ethnic study of atherosclerosis (MESA). Eur Heart J. 2018 Jul 1;39(25):2401–2408. 10.1093/eurheartj/ehy217 PMC6030975 29688297

[24] Stern MP, Williams K, Haffner SM Identification of persons at high risk for type 2 diabetes mellitus: do we need the oral glucose tolerance test? Ann Intern Med 12002; 36 10.7326/0003-4819-136-8-200204160-00006 11955025

[25] Levine ME, Lu AT, Quach A, Chen BH, Assimes TL, Bandinelli S, Hou L, Baccarelli AA, Stewart JD, Li Y, Whitsel EA, Wilson JG, Reiner AP, Aviv A, Lohman K, Liu Y, Ferrucci L, Horvath S. An epigenetic biomarker of aging for lifespan and healthspan. Aging (Albany NY). 2018;10:573–91. 29676998 10.18632/aging.101414 PMC5940111

[26] Roffman CE, Buchanan J, Allison GT. Charlson comorbidities index J Physiother. 2016;62:171. 27298055 10.1016/j.jphys.2016.05.008

[27] Faul F, Erdfelder E, Buchner A, Lang AG G* Power (Version 3.1. 9.2). Germany: University of Kiel (2014)

[28] Beaudreau SA, Spira AP, Stewart A, Kezirian EJ, Lui LY, Ensrud K, Redline S, Ancoli-Israel S, Stone KL; Study of Osteoporotic Fractures. Validation of the Pittsburgh Sleep Quality Index and the Epworth Sleepiness Scale in older black and white women. Sleep Med 13 2012:36-42 10.1016/j.sleep.2011.04.005 PMC3586742 22033120

[29] Yang PY, Ho KH, Chen HC, Chien MY. Exercise training improves sleep quality in middle-aged and older adults with sleep problems: a systematic review. J Physiother. 2012;58:157–63. 22884182 10.1016/S1836-9553(12)70106-6

[30] Vargas PA, Flores M, Robles E. Sleep quality and body mass index in college students: the role of sleep disturbances. J Am Coll Health. 2014;62:534–41. 24933244 10.1080/07448481.2014.933344 PMC4221412

[31] Rahe C, Czira ME, Teismann H, Berger K. Associations between poor sleep quality and different measures of obesity. Sleep Med. 2015;16:1225–8. 26429750 10.1016/j.sleep.2015.05.023

[32] Spiegel K, Leproult R, van Cauter E. Impact of sleep debt on metabolic and endocrine function. Lancet. 1999;354:1435–9. 10543671 10.1016/S0140-6736(99)01376-8

[33] Zimberg IZ, Dâmaso A, Del Re M, Carneiro AM, de Sá SH, de Lira F, et al. Short sleep duration and obesity: mechanisms and future perspectives. Cell Biochem Funct. 2012;30:524–9. 22473743 10.1002/cbf.2832

[34] Ware JE, Snow KK, Kosinski M, Gandek B (1993) SF-36 health survey. Manual and interpretation guide

[35] Beaufrère B, Morio B. Fat and protein redistribution with aging: metabolic considerations. Eur J Clin Nutr. 2000;54:S48-53. 11041075 10.1038/sj.ejcn.1601025

[36] Yuan L, Chang M, Wang J. Abdominal obesity, body mass index and the risk of frailty in community-dwelling older adults: a systematic review and meta-analysis. Age Ageing. 2021;50:1118–28. 33693472 10.1093/ageing/afab039

[37] Reilly SM, Saltiel AR. Adapting to obesity with adipose tissue inflammation. Nat Rev Endocrinol. 2017;13:633–43. 28799554 10.1038/nrendo.2017.90

[38] Zoico E, Rossi A, Di Francesco V, Sepe A, Olioso D, Pizzini F, Fantin F, Bosello O, Cominacini L, Harris TB, Zamboni M. Adipose tissue infiltration in skeletal muscle of healthy elderly men: relationships with body composition, insulin resistance, and inflammation at the systemic and tissue level. J Gerontol A Biol Sci Med Sci. 2010;65:295–9. 19864639 10.1093/gerona/glp155 PMC4051307

[39] Muoio DM. Metabolic inflexibility: when mitochondrial indecision leads to metabolic gridlock. Cell. 2014;159:1253–62. 25480291 10.1016/j.cell.2014.11.034 PMC4765362

[40] Taniguchi M, Yamada Y, Fukumoto Y, Sawano S, Minami S, Ikezoe T, Watanabe Y, Kimura M, Ichihashi N. Increase in echo intensity and extracellular-to-intracellular water ratio is independently associated with muscle weakness in elderly women. Eur J Appl Physiol. 2017;117:2001–7. 28755131 10.1007/s00421-017-3686-x

[41] Yamada Y, Yoshida T, Yokoyama K, Watanabe Y, Miyake M, Yamagata E, Yamada M, Kimura M; Kyoto-Kameoka Study The extracellular to intracellular water ratio in upper legs is negatively associated with skeletal muscle strength and gait speed in older people. J Gerontol A BiolSci Med Sci 72 2017:293-298 10.1093/gerona/glw125 27422438

[42] Cruz-Jentoft AJ, Bahat G, Bauer J, Boirie Y, Bruyère O, Cederholm T, Cooper C, Landi F, Rolland Y, Sayer AA, Schneider SM, Sieber CC, Topinkova E, Vandewoude M, Visser M, Zamboni M; Writing Group for the European Working Group on Sarcopenia in Older People 2 (EWGSOP2), and the Extended Group for EWGSOP2 (2019) Sarcopenia: revised European consensus on definition and diagnosis. Age Ageing 48:16-31 10.1093/ageing/afy169 PMC6322506 30312372

[43] Serra-Prat M, Lorenzo I, Palomera E, Yébenes JC, Campins L, Cabré M. Intracellular water content in lean mass is associated with muscle strength, functional capacity, and frailty in community-dwelling elderly individuals. A cross-sectional study Nutrients. 2019;11:661. 30893821 10.3390/nu11030661 PMC6471552

[44] Sawka MN, Convertino VA, Eichner ER, Schnieder SM, Young AJ. Blood volume: importance and adaptations to exercise training, environmental stresses, and trauma/sickness. Med Sci Sports Exerc. 2000;32:332–48. 10694114 10.1097/00005768-200002000-00012

[45] Hu M, Lin W. Effects of exercise training on red blood cell production: implications for anemia. Acta Haematol. 2012;127:156–64. 22301865 10.1159/000335620

[46] Ruggiero C, Metter EJ, Cherubini A, Maggio M, Sen R, Najjar SS, Windham GB, Ble A, Senin U, Ferrucci L. White blood cell count and mortality in the Baltimore Longitudinal Study of Aging. J Am Coll Cardiol. 2007;49:1841–50. 17481443 10.1016/j.jacc.2007.01.076 PMC2646088

[47] van Holten TC, Waanders LF, de Groot PG, Vissers J, Hoefer IE, Pasterkamp G, Prins MW, Roest M. Circulating biomarkers for predicting cardiovascular disease risk; a systematic review and comprehensive overview of meta-analyses. PLoS ONE. 2013;8:e62080. 23630624 10.1371/journal.pone.0062080 PMC3632595

[48] Lee LE, Pyo JY, Ahn SS, Song JJ, Park YB, Lee SW. Clinical significance of large unstained cell count in estimating the current activity of antineutrophil cytoplasmic antibody-associated vasculitis. Int J Clin Pract. 2021;75:e14512. 34118131 10.1111/ijcp.14512

[49] Stepanova M, Rodriguez E, Birerdinc A, Baranova A. Age-independent rise of inflammatory scores may contribute to accelerated aging in multi-morbidity. Oncotarget. 2015;6:1414–21. 25638154 10.18632/oncotarget.2725 PMC4359303

[50] Andrews R, Greenhaff P, Curtis S, et al. The effect of dietary creatine supplementation on skeletal muscle metabolism in congestive heart failure. Eur Heart J. 1998;19:617–22. 9597411 10.1053/euhj.1997.0767

[51] Thongprayoon C, Cheungpasitporn W, Kashani K. Serum creatinine level, a surrogate of muscle mass, predicts mortality in critically ill patients. J Thorac Dis. 2016;8:E305-311. 27162688 10.21037/jtd.2016.03.62 PMC4842835

[52] Schutte JE, Longhurst JC, Gaffney FA, Bastian BC, Blomqvist CG. Total plasma creatinine: an accurate measure of total striated muscle mass. J Appl Physiol Respir Environ Exerc Physiol. 1981;51:762–6. 7327978 10.1152/jappl.1981.51.3.762

[53] Diago CAA, Señaris JAA. Should we pay more attention to low creatinine levels? Endocrinol Diabetes Nutr (Engl Ed). 2020;67:486–92. 32331974 10.1016/j.endinu.2019.12.008

[54] Hunter CA, Jones SA. IL-6 as a keystone cytokine in health and disease. Nat Immunol. 2015;16:448–57. 25898198 10.1038/ni.3153

[55] Pedersen BK, Steensberg A, Schjerling P. Exercise and interleukin-6. Curr Opin Hematol. 2001;8:137–41. 11303145 10.1097/00062752-200105000-00002

[56] Jonsdottir IH, Schjerling P, Ostrowski K, Asp S, Richter EA, Pedersen BK. Muscle contractions induce interleukin-6 mRNA production in rat skeletal muscles. J Physiol. 2000;528(Pt 1):157–63. 11018114 10.1111/j.1469-7793.2000.00157.x PMC2270126

[57] Steensberg A, van Hall G, Osada T, Sacchetti M, Saltin B, Klarlund Pedersen B. Production of interleukin-6 in contracting human skeletal muscles can account for the exercise-induced increase in plasma interleukin-6. J Physiol. 2000;529(Pt 1):237–42. 11080265 10.1111/j.1469-7793.2000.00237.x PMC2270169

[58] Lavie CJ, McAuley PA, Church TS, Milani RV, Blair SN. Obesity and cardiovascular diseases: implications regarding fitness, fatness, and severity in the obesity paradox. J Am Coll Cardiol. 2014;63:1345–54. 24530666 10.1016/j.jacc.2014.01.022

