# Supplementary material for: Fitness age outperforms body mass index in differentiating aging patterns and health risk profiles of healthy adults aged 51–80 years
Source: GeroScience. 2024 Mar 18;46(6):5875–90. doi: 10.1007/s11357-024-01125-z (PMC11493939; doi:10.1007/s11357-024-01125-z)
Supplement: Supplementary file 1 — Supplementary file1 (DOCX 67 KB) [file 11357_2024_1125_MOESM1_ESM.docx]

| **Supplementary Material** 1 Health-related outcomes according to FitAA status. | | | | | | | | |
| --- | --- | --- | --- | --- | --- | --- | --- | --- |
| **Variable** | | **FitAA status** | | | | **Statistics** | | |
|  |  | **Total**  **(*n*=176)** | **Decelerated**  **(*n*=43)** | **Normal**  **(*n*=97)** | **Accelerated**  **(*n*=36)** | **FitAA main effect** | | **Post Hoc Multiple Comparisons** |
|  |  |  |  |  |  | **F value** | **P value** |  |
| **MOCA (score)** | **Whole sample** | **23.89±3.46** | **23.93±3.31** | **24.18±3.43** | **23.03±3.69** | 1.362 | 0.259 | Decel>Accel p=0.783  Decel<Normal p=1.00  Normal>Accel p=0.303 |
|  | Women | 23.56±3.51 | 23.53±4.43 | 23.94±3.15 | 22.55±3.57 | 1.318 | 0.271 |  |
|  | Men | 23.89±3.46 | 24.5±2.09 | 24.64±3.91 | 24.0±3.9 | 0.174 | 0.841 |  |
|  | FitAA*Sex |  |  |  |  | 0.14 | 0.87 | - |
| **GDS (score)** | **Whole sample** | **4.39±2.71** | **4.09±3.05** | **4.44±2.55** | **4.65±2.73** | 0.424 | 0.655 | Decel<Accel p=0.783  Decel<Normal p=1.00  Normal<Accel p=0.303 |
|  | Women | 4.61±2.69 | 4.53±3.1 | 4.75±2.64 | 4.27±2.55 | 0.26 | 0.771 |  |
|  | Men | 4.07±2.72 | 3.75±3.04 | 3.85±2.28 | 5.33±3.03 | 1.589 | 0.207 |  |
|  | FitAA*Sex |  |  |  |  | 1.583 | 0.208 | - |
| **SF-36 Role limitations due to physical problems** | **Whole sample** | **74.27±32.47** | **80.81±28.78** | **72.87±32.52** | **69.85±36.30** | 1.279 | 0.281 | Decel>Accel p=0.428  Decel>Normal p=0.555  Normal>Accel p=1.00 |
|  | Women | 74.26±31.94 | 88.16±24.11 | 72.54±31.19 | 67.05±37.31 | 2.365 | 0.097 |  |
|  | Men | 74.28±33.48 | 75.0±31.28 | 73.48±35.32 | 75.0±35.35 | 0.019 | 0.981 |  |
|  | FitAA*Sex |  |  |  |  | 1.071 | 0.34 | - |
| **SF-36 Bodily Pain** | **Whole sample** | **72.42±23.81** | **78.49±22.19** | **71.14±23.71** | **68.38±25.28** | 2.049 | 0.132 | Decel>Accel p=0.193  Decel>Normal p=0.277  Normal>Accel p=1.00 |
|  | Women | 70.63±23.8 | 80.26±20.63 | 70.97±23.37 | 61.36±25.04 | 3.321 | 0.039 |  |
|  | Men | 75.12±23.74 | 77.08±23.70 | 71.47±24.73 | 81.25±20.98 | 0.892 | 0.412 |  |
|  | FitAA*Sex |  |  |  |  | 2.497 | 0.08 | - |
| **SF-36 General health perceptions** | **Whole sample** | **63.47±13.81** | **66.28±13.810** | **62.50±13.24** | **62.65±15.29** | 1.189 | 0.307 | Decel>Accel p=0.759  Decel>Normal p=0.412  Normal<Accel p=1.00 |
|  | Women | 62.26±14.51 | 66.05±14.58 | 61.03±13.77 | 62.5±16.53 | 0.965 | 0.383 |  |
|  | Men | 65.29±13.81 | 66.46±13.47 | 65.30±11.85 | 62.92±13.39 | 0.262 | 0.770 |  |
|  | FitAA*Sex |  |  |  |  | 0.387 | 0.68 | - |
| **SF-36 Role limitations due to emotional problems** | **Whole sample** | **65.32±38.26** | **75.19±37.86** | **61.12±37.97** | **64.71±38.44** | 2.037 | 0.134 | Decel>Accel p=0.694  Decel>Normal p=0.136  Normal<Accel p=1.00 |
|  | Women | 58.67±39.81 | 75.44±42.81 | 52.93±38.62 | 60.61±37.99 | 2.692 | 0.071 |  |
|  | Men | 75.36±33.65 | 75.0±34.40 | 76.77±31.72 | 72.22±39.78 | 0.067 | 0.935 |  |
|  | FitAA*Sex |  |  |  |  | 1.546 | 0.216 | - |
| **SF-36 Mental health** | **Whole sample** | **71.61±15.65** | **74.23±16.03** | **70.17±16.20** | **72.35±13.37** | 1.051 | 0.352 | Decel>Accel p=1.00  Decel>Normal p=0.476  Normal<Accel p=1.00 |
|  | Women | 69.0±16.16 | 73.26±16.92 | 67.75±16.58 | 68.90±14.23 | 0.933 | 0.395 |  |
|  | Men | 75.54±14.07 | 75.0±15.61 | 74.79±14.58 | 78.67±9.08 | 0.30 | 0.741 |  |
|  | FitAA*Sex |  |  |  |  | 0.682 | 0.507 | - |
| All data are scores, which are shown as mean ± standard deviation. *Abbreviations: FitAA, Fitness Age Acceleration; MOCA, Montreal Cognitive Assessment; decel, decelerated status at FitAA; accel, accelerated status at FitAA; normal, normal status at FitAA; GDS, Geriatric Depression Scale; SF-36, 36-Item Short* Form Survey. | | | | | | | | |

| **Supplementary Material 2** Nutritional and anthropometric data according to FitAA status. | | | | | | | | | | | |
| --- | --- | --- | --- | --- | --- | --- | --- | --- | --- | --- | --- |
| **Variable** |  | **FitAA status** | | | | | | | **Statistics** | | |
|  |  | **Total**  (*n*=176) | | **Decelerated**  (*n*=43) | | **Normal**  (*n*=97) | | **Accelerated**  (*n*=36) | **FitAA main effect** | | **Post Hoc Multiple Comparisons** |
|  |  |  |  |  |  |  |  |  | **F value** | **P value** |  |
| **GPC – milk and derivates** | **Whole sample** | **18.11±14.51** | | **20.78±15.14** | | **17.29±13.93** | | **16.76±15.31** | 0.954 | 0.388 | Decel>Accel p=0.766  Decel>Normal p=0.626  Normal>Accel p=1.00 |
|  | Women | 19.63±15.97 | | 22.89±18.76 | | 18.29±14.97 | | 20.56±16.45 | 0.739 | 0.48 |  |
|  | Men | 15.9±11.85 | | 19.13±11.77 | | 15.38±11.67 | | 10.55±11.33 | 1.352 | 0.262 |  |
|  | FitAA*Sex |  | |  | |  | |  | 0.627 | 0.53 | - |
| **GPC – tubers** | **Whole sample** | **64.62±22.13** | | **65.32±18.98** | | **63.46±23.68** | | **67.03±22.06** | 0.307 | 0.736 | Decel<Accel p=1.00  Decel>Normal p=1.00  Normal<Accel p=1.00 |
|  | Women | 60.54±22.52 | | 56.67±20.13 | | 61.39±23.49 | | 61.78±22.46 | 0.356 | 0.701 |  |
|  | Men | 70.57±20.3 | | 72.09±15.26 | | 67.45±23.95 | | 75.64±19.32 | 0.651 | 0.523 |  |
|  | FitAA*Sex |  | |  | |  | |  | 0.732 | 0.48 | - |
| **GPC – fruits** | **Whole sample** | **73.81±26.95** | | **75.10±22.54** | | **74.39±29.29** | | **70.31±26.08** | 0.308 | 0.735 | Decel>Accel p=1.00  Decel>Normal p=1.00  Normal>Accel p=1.00 |
|  | Women | 75.1±26.15 | | 78.94±18.61 | | 75.88±27.94 | | 68.83±26.99 | 0.679 | 0.509 |  |
|  | Men | 71.94±28.18 | | 72.09±25.2 | | 71.52±32.03 | | 72.72±25.59 | 0.008 | 0.992 |  |
|  | FitAA*Sex |  | |  | |  | |  | 0.338 | 0.71 | - |
| **GPC – meat, fish, eggs** | **Whole sample** | **30.30±11.91** | | **30.05±10.90** | | **29.8±12.13** | | **32.14±12.82** | 0.426 | 0.654 | Decel<Accel p=1.00  Decel>Normal p=1.00  Normal<Accel p=1.00 |
|  | Women | 29.38±11.6 | | 27.39±9.66 | | 29.73±11.97 | | 30.28±12.52 | 0.324 | 0.724 |  |
|  | Men | 31.65±12.32 | | 32.13±11.56 | | 29.93±12.64 | | 35.18±13.31 | 0.797 | 0.452 |  |
|  | FitAA*Sex |  | |  | |  | |  | 0.666 | 0.51 | - |
| **Body circumfer –**  **brachial (cm)** | **Whole sample** | **31.92±3.20** | | **31.55±3.24** | | **31.79±3.21** | | **32.73±3.06** | 1.477 | 0.231 | Decel<Accel p=0.325  Decel<Normal p=1.00  Normal<Accel p=0.416 |
|  | Women | 31.43±3.19 | | 29.59±2.68 | | 31.4±3.18 | | 33.11±2.8 | 6.758 | 0.002 |  |
|  | Men | 32.63±3.1 | | 33.11±2.80 | | 32.5±3.19 | | 32.05±3.52 | 0.541 | 0.583 |  |
|  | FitAA*Sex |  | |  | |  | |  | 5.147 | 0.01 | - |
| **Body circumfer –**  **waist (cm)** | **Whole sample** | **88.61±11.58** | | **87.75±11.11** | | **87.89±12.04** | | **91.73±10.44** | 1.557 | 0.214 | Decel<Accel p=0.401  Decel<Normal p=1.00  Normal<Accel p=0.289 |
|  | Women | 83.71±10.3 | | 79.42±9.22 | | 83.36±10.05 | | 88.4±10.43 | 4.417 | 0.014 |  |
|  | Men | 95.82±9.36 | | 94.35±7.5 | | 96.15±11.05 | | 97.84±7.52 | 0.55 | 0.578 |  |
|  | FitAA*Sex |  | |  | |  | |  | 0.710 | 0.49 | - |
| **Body circumfer –**  **calf (cm)** | **Whole sample** | **37.54±2.93** | | **37.36±2.91** | | **37.55±2.98** | | **37.74±2.89** | 0.156 | 0.856 | Decel<Accel p=1.00  Decel<Normal p=1.00  Normal<Accel p=1.00 |
|  | Women | 36.83±2.75 | | 35.77±2.5 | | 36.99±2.85 | | 37.3±2.52 | 1.743 | 0.178 |  |
|  | Men | 38.59±2.89 | | 38.63±2.62 | | 38.58±2.97 | | 38.55±3.44 | 0.003 | 0.997 |  |
|  | FitAA*Sex |  |  | |  | |  | | 0.936 | 0.394 | - |
| All data are scores, which are shown as mean ± standard deviation. *Abbreviations: FitAA, Fitness Age Acceleration; GPC,” Griglia Porzioni Consumate*” (Grid Portions Consumed); *cm*, centimeters. | | | | | | | | | | | |

| **Supplementary Material 3** Hematochemical parameters according to FitAA status. | | | | | | | | |
| --- | --- | --- | --- | --- | --- | --- | --- | --- |
| **Variable** | | **FitAA status** | | | | **Statistics** | | |
|  |  | **Total**  (*n*=176) | **Decelerated**  (*n*=43) | **Normal**  (*n*=97) | **Accelerated**  (*n*=36) | **FitAA main effect** | | **Post Hoc Multiple Comparisons** |
|  |  |  |  |  |  | **F value** | **P value** |  |
| **RBC (x10^3/μL)** | **Whole sample** | **5.05±0.55** | **5.16±0.60** | **5.02±0.52** | **5.02±0.55** | 0.960 | 0.385 | Decel>Accel p=0.062  Decel>Normal p=1.00  Normal<Accel p=0.084 |
|  | Women | 4.91±0.49 | 4.77±0.38 | 4.93±0.50 | 4.96±0.55 | 0.874 | 0.419 |  |
|  | Men | 5.28±0.56 | 5.48±0.56 | 5.19±0.53 | 5.12±0.56 | 2.665 | 0.073 |  |
|  | FitAA*Sex |  |  |  |  | 3.312 | 0.04 | *Women*  Decel>Normal p=0.049 |
| **MCV (μm^3^)** | **Whole sample** | **87.26±9.47** | **88.33±8.08** | **86.83±10.13** | **87.14±9.33** | 0.365 | 0.695 | Decel<Accel p=1.00  Decel>Normal p=1.00  Normal<Accel p=1.00 |
|  | Women | 87.02±9.31 | 89.91±5.44 | 86.74±10.16 | 85.24±9.24 | 1.273 | 0.283 |  |
|  | Men | 89.63±9.76 | 87.03±9.67 | 86.99±10.22 | 90.47±8.88 | 0.655 | 0.521 |  |
|  | FitAA*Sex |  |  |  |  | 1.610 | 0.20 | - |
| **MCH**  **(10 × [Hb/RBC])** | **Whole sample** | **27.83±3.57** | **28.34±3.24** | **27.58±3.76** | **27.88±3.46** | 0.655 | 0.521 | Decel<Accel p=1.00  Decel>Normal p=0.766  Normal<Accel p=1.00 |
|  | Women | 27.67±3.45 | 28.84±2.16 | 27.55±3.75 | 26.97±3.37 | 1.477 | 0.231 |  |
|  | Men | 28.08±3.75 | 27.93±3.91 | 27.65±3.83 | 29.48±3.12 | 1.179 | 0.31 |  |
|  | FitAA*Sex |  |  |  |  | 2.112 | 0.12 | - |
| **MCHC (g/dL)** | **Whole sample** | **31.88±1.11** | **32.01±1.22** | **31.80±1.09** | **31.93±1.06** | 0.541 | 0.589 | Decel>Accel p=1.00  Decel>Normal p=0.969  Normal<Accel p=1.00 |
|  | Women | 31.81±1.02 | 32.04±0.85 | 31.83±1.1 | 31.58±0.93 | 0.872 | 0.420 |  |
|  | Men | 31.98±1.24 | 31.98±1.47 | 31.75±1.1 | 32.56±1.01 | 2.307 | 0.103 |  |
|  | FitAA*Sex |  |  |  |  | 2.755 | 0.07 | - |
| **RDW (%)** | **Whole sample** | **13.79±1.10** | **13.73±1.13** | **13.79±1.09** | **13.88±1.09** | 0.165 | 0.848 | Decel<Accel p=1.00  Decel<Normal p=1.00  Normal<Accel p=1.00 |
|  | Women | 13.79±1.06 | 13.43±0.54 | 13.82±1.14 | 14.0±1.13 | 1.420 | 0.245 |  |
|  | Men | 13.8±1.16 | 13.97±1.41 | 13.71±1.01 | 13.66±1.04 | 0.476 | 0.622 |  |
|  | FitAA*Sex |  |  |  |  | 1.725 | 0.18 | - |
| **HDW (mg/dL)** | **Whole sample** | **2.49±0.35** | **2.47±0.30** | **2.48±0.36** | **2.57±0.40** | 1.019 | 0.363 | Decel<Accel p=0.626  Decel<Normal p=1.00  Normal<Accel p=0.565 |
|  | Women | 2.45±0.36 | 2.32±0.21 | 2.46±0.39 | 2.51±0.39 | 1.515 | 0.223 |  |
|  | Men | 2.6±0.36 | 2.59±0.31 | 2.57±0.38 | 2.68±0.42 | 0.418 | 0.66 |  |
|  | FitAA*Sex |  |  |  |  | 0.692 | 0.502 | - |
| **PLT (x10^9/L)** | **Whole sample** | **229.98±51.60** | **218.29±42.50** | **233.27±48.08** | **235.67±68.42** | 1.474 | 0.232 | Decel<Accel p=0.445  Decel<Normal p=0.359  Normal<Accel p=1.00 |
|  | Women | 239.72±43.78 | 229.26±47.32 | 241.1±43.5 | 245.19±42.84 | 0.549 | 0.579 |  |
|  | Men | 215.06±58.76 | 209.22±36.67 | 217.87±53.46 | 219.0±99.02 | 0.237 | 0.790 |  |
|  | FitAA*Sex |  |  |  |  | 0.033 | 0.97 | - |
| **MPV (fL)** | **Whole sample** | **8.20±0.93** | **8.10±0.92** | **8.22±0.88** | **8.27±1.08** | 0.329 | 0.720 | Decel<Accel p=1.00  Decel<Normal p=1.00  Normal<Accel p=1.00 |
|  | Women | 8.16±0.92 | 8.02±1.01 | 8.21±0.86 | 8.13±1.06 | 0.303 | 0.739 |  |
|  | Men | 8.26±0.95 | 8.17±0.86 | 8.23±0.95 | 8.5±1.13 | 0.514 | 0.599 |  |
|  | FitAA*Sex |  |  |  |  | 0.384 | 0.68 | - |
| **Neutrophils (%)** | **Whole sample** | **55.02±8.08** | **54.95±7.74** | **54.50±8.51** | **56.57±7.22** | 0.796 | 0.453 | Decel<Accel p=1.00  Decel>Normal p=1.00  Normal<Accel p=0.628 |
|  | Women | 53.67±8.23 | 51.63±7.67 | 53.29±8.5 | 56.62±7.42 | 2.162 | 0.118 |  |
|  | Men | 57.06±7.46 | 57.69±6.79 | 56.82±8.18 | 56.47±7.16 | 0.121 | 0.886 |  |
|  | FitAA*Sex |  |  |  |  |  |  | - |
| **Lymphocytes (%)** | **Whole sample** | **33.26±7.74** | **33.47±7.33** | **33.83±8.13** | **31.40±7.00** | 1.230 | 0.295 | Decel>Accel p=0.753  Decel<Normal p=1.00  Normal>Accel p=0.365 |
|  | Women | 34.88±7.53 | 37.16±6.86 | 35.26±7.66 | 31.69±6.99 | 2.891 | 0.058 |  |
|  | Men | 30.83±7.46 | 30.41±6.33 | 31.11±8.42 | 30.88±7.29 | 0.059 | 0.943 |  |
|  | FitAA*Sex |  |  |  |  | 1.399 | 0.25 | - |
| **Monocytes (%)** | **Whole sample** | **5.93±1.33** | **6.09±1.61** | **5.90±1.31** | **5.84±1.01** | 0.426 | 0.654 | Decel>Accel p=1.00  Decel>Normal p=1.00  Normal>Accel p=1.00 |
|  | Women | 5.71±1.27 | 6.0±1.53 | 5.64±1.28 | 5.67±0.98 | 0.567 | 0.569 |  |
|  | Men | 6.27±1.37 | 6.17±1.7 | 6.4±1.23 | 6.13±1.03 | 0.273 | 0.761 |  |
|  | FitAA*Sex |  |  |  |  | 0.707 | 0.495 | - |
| **Basophils (%)** | **Whole sample** | **0.69±0.27** | **0.69±0.27** | **0.68±0.28** | **0.71±0.27** | 0.233 | 0.793 | Decel<Accel p=1.00  Decel>Normal p=1.00  Normal<Accel p=1.00 |
|  | Women | 0.72±0.3 | 0.73±0.28 | 0.71±0.3 | 0.74±0.3 | 0.116 | 0.89 |  |
|  | Men | 0.64±0.25 | 0.66±0.26 | 0.62±0.24 | 0.68±0.24 | 0.251 | 0.778 |  |
|  | FitAA*Sex |  |  |  |  | 0.032 | 0.97 | - |
| **Neutrophils (x10^3/μL)** | **Whole sample** | **3.30±1.01** | **3.21±1.05** | **3.22±0.94** | **3.65±1.10** | 2.412 | 0.093 | Decel<Accel p=0.189  Decel<Normal p=1.00  Normal<Accel p=0.116 |
|  | Women | 3.06±0.9 | 2.82±1.02 | 3.02±0.76 | 3.4±1.08 | 1.945 | 0.146 |  |
|  | Men | 3.66±1.07 | 3.53±0.97 | 3.60±1.13 | 4.08±1.04 | 1.383 | 0.254 |  |
|  | FitAA*Sex |  |  |  |  | 0.072 | 0.93 | - |
| **Lymphocytes (x10^3/μL)** | **Whole sample** | **1.95±0.56** | **1.90±0.47** | **1.96±0.55** | **2.00±0.70** | 0.300 | 0.741 | Decel<Accel p=1.00  Decel<Normal p=1.00  Normal<Accel p=1.00 |
|  | Women | 1.94±0.51 | 1.95±0.46 | 1.98±0.54 | 1.82±0.44 | 0.611 | 0.544 |  |
|  | Men | 1.97±0.64 | 1.86±0.49 | 1.92±0.58 | 2.32±0.94 | 2.849 | 0.061 |  |
|  | FitAA*Sex |  |  |  |  | 3.107 | 0.47 | - |
| **Monocytes (x10^3/μL)** | **Whole sample** | **0.35±0.10** | **0.34±0.10** | **0.35±0.10** | **0.37±0.10** | 0.695 | 0.501 | Decel<Accel p=0.823  Decel<Normal p=1.00  Normal<Accel p=0.891 |
|  | Women | 0.33±0.08 | 0.32±0.09 | 0.32±0.08 | 0.35±0.09 | 1.095 | 0.337 |  |
|  | Men | 0.39±0.11 | 0.37±0.1 | 0.41±0.11 | 0.41±0.12 | 1.214 | 0.30 |  |
|  | FitAA*Sex |  |  |  |  | 0.536 | 0.58 | - |
| **Basophils (x10^3/μL)** | **Whole sample** | **0.04±0.09** | **0.04±0.05** | **0.05±0.11** | **0.05±0.05** | 0.237 | 0.789 | Decel<Accel p=1.00  Decel<Normal p=1.00  Normal>Accel p=1.00 |
|  | Women | 0.05±0.11 | 0.04±0.05 | 0.06±0.13 | 0.05±0.05 | 0.374 | 0.689 |  |
|  | Men | 0.32±0.05 | 0.03±0.05 | 0.02±0.04 | 0.05±0.05 | 0.350 | 0.705 |  |
|  | FitAA*Sex |  |  |  |  | 0.606 | 0.54 | - |
| **Glycemia (mg/dL)** | **Whole sample** | **93.99±12.10** | **96.20±10.65** | **94.00±12.82** | **91.13±11.37** | 1.544 | 0.217 | Decel>Accel p=0.24  Decel>Normal p=1.00  Normal>Accel p=0.758 |
|  | Women | 92.42±11.1 | 93.79±9.29 | 92.56±13.43 | 90.7±9.68 | 0.332 | 0.718 |  |
|  | Men | 96.45±11.93 | 98.38±11.53 | 96.75±11.25 | 91.91±14.44 | 1.066 | 0.35 |  |
|  | FitAA*Sex |  |  |  |  | 0.197 | 0.82 | - |
| **Urea (mg/dL)** | **Whole sample** | **36.93±9.80** | **40.12±9.87** | **35.11±8.78** | **38.06±11.49** | 4.198 | 0.017 | Decel>Accel p=1.00  Decel>Normal p=0.017  Normal<Accel p=0.408 |
|  | Women | 35.63±9.64 | 40.32±11.54 | 33.54±7.58 | 37.55±11.69 | 4.139 | 0.018 |  |
|  | Men | 38.88±9.79 | 39.96±8.53 | 38.09±10.16 | 38.92±11.61 | 0.254 | 0.776 |  |
|  | FitAA*Sex |  |  |  |  | 0.998 | 0.37 | - |
| **Total protein (g/dL)** | **Whole sample** | **7.06±0.42** | **7.09±0.42** | **7.04±0.39** | **7.10±0.50** | 0.306 | 0.737 | Decel<Accel p=1.00  Decel>Normal p=1.00  Normal<Accel p=1.00 |
|  | Women | 7.07±0.41 | 7.04±0.49 | 7.04±0.38 | 7.17±0.42 | 0.756 | 0.471 |  |
|  | Men | 7.06±0.43 | 7.13±0.36 | 7.05±0.40 | 6.98±0.61 | 0.501 | 0.607 |  |
|  | FitAA*Sex |  |  |  |  | 0.944 | 0.39 | - |
| **Albumina (g/dL)** | **Whole sample** | **4.25±0.23** | **4.28±0.22** | **4.24±0.22** | **4.25±0.24** | 0.654 | 0.522 | Decel>Accel p=1.00  Decel>Normal p=0.767  Normal<Accel p=0.1.00 |
|  | Women | 4.24±0.22 | 4.24±0.22 | 4.24±0.22 | 4.25±0.20 | 0.017 | 0.983 |  |
|  | Men | 4.26±0.24 | 4.32±0.22 | 4.22±0.23 | 4.24±0.3 | 1.346 | 0.263 |  |
|  | FitAA*Sex |  |  |  |  | 0.786 | 0.46 | - |
| **ALP (U/L)** | **Whole sample** | **76.47±21.33** | **73.57±19.47** | **76.19±22.50** | **81.09±19.97** | 1.150 | 0.319 | Decel<Accel p=0.403  Decel<Normal p=1.00  Normal<Accel p=0.791 |
|  | Women | 77.51±18.22 | 75.37±15.51 | 76.23±18.13 | 83.45±20.42 | 0.97 | 0.381 |  |
|  | Men | 74.92±25.34 | 72.09±22.47 | 76.12±29.42 | 77.17±19.42 | 0.317 | 0.729 |  |
|  | FitAA*Sex |  |  |  |  | 0.249 | 0.78 | - |
| **Serum iron (μg/dL)** | **Whole sample** | **91.36±27.79** | **96.17±27.80** | **90.26±26.84** | **99.34±30.49** | 0.874 | 0.419 | Decel<Accel p=0.703  Decel>Normal p=0.778  Normal<Accel p=1.00 |
|  | Women | 86.86±25.19 | 95.0±24.72 | 86.58±24.7 | 79.95±26.10 | 1.481 | 0.23 |  |
|  | Men | 98.11±30.24 | 97.18±30.75 | 97.16±29.63 | 102.33±33.17 | 0.175 | 0.84 |  |
|  | FitAA*Sex |  |  |  |  | 1.177 | 0.31 | - |
| **IGF-1 (ng/ml)** | **Whole sample** | **0.77±0.78** | **0.87±0.86** | **0.76±0.83** | **0.69±0.51** | 0.506 | 0.604 | Decel>Accel p=0.990  Decel>Normal p=1.00  Normal>Accel p=1.00 |
|  | Women | 0.79±0.8 | 1.03±1.08 | 0.76±081 | 0.67±0.44 | 0.984 | 0.376 |  |
|  | Men | 0.75±0.75 | 0.76±0.7 | 0.75±0.87 | 0.72±0.63 | 0.01 | 0.99 |  |
|  | FitAA*Sex |  |  |  |  | 0.416 | 0.66 | - |
| **Leptin (ng/mL)** | **Whole sample** | **458.17±360.02** | **520.96±441.02** | **440.53±305.26** | **427.98±390.25** | 0.865 | 0.423 | Decel>Accel p=0.807  Decel>Normal p=0.695  Normal>Accel p=1.00 |
|  | Women | 474.98±376.93 | 495.06±446.22 | 466.39±322.55 | 481.75±467.35 | 0.05 | 0.951 |  |
|  | Men | 432.83±334.07 | 542.35±445.53 | 391.23±267.13 | 333.88±175.36 | 1.722 | 0.182 |  |
|  | FitAA*Sex |  |  |  |  | 0.703 | 0.5 | - |
| **PSH**  **(μmol/g protein)** | **Whole sample** | **4.82±1.00** | **5.08±0.69** | **4.73±1.15** | **4.73±0.82** | 1.935 | 0.148 | Decel>Accel p=0.398  Decel>Normal p=0.184  Normal>Accel p=1.00 |
|  | Women | 4.75±1.06 | 4.78±0.76 | 4.79±1.24 | 4.59±0.7 | 0.351 | 0.704 |  |
|  | Men | 4.93±0.9 | 5.34±0.52 | 4.63±0.98 | 4.99±0.99 | 3.44 | 0.034 |  |
|  | FitAA*Sex |  |  |  |  | 2.18 | 0.12 | - |
| **MDA (μmol/L)** | **Whole sample** | **2.64±1.30** | **2.84±1.31** | **2.49±1.23** | **2.80±1.46** | 1.330 | 0.267 | Decel>Accel p=0.457  Decel>Normal p=0.626  Normal<Accel p=0.752 |
|  | Women | 2.34±1.30 | 2.35±0.93 | 2.28±1.34 | 2.49±1.51 | 0.219 | 0.803 |  |
|  | Men | 3.09±1.17 | 3.25±1.45 | 2.89±0.89 | 3.33±1.25 | 0.824 | 0.44 |  |
|  | FitAA*Sex |  |  |  |  | 0.223 | 0.8 | - |
| **PON (U/L)** | **Whole sample** | **157.82±93.84** | **169.00±103.16** | **156.31±87.39** | **147.50±101.02** | 0.487 | 0.615 | Decel>Accel p=1.00  Decel>Normal p=1.00  Normal>Accel p=1.00 |
|  | Women | 153.71±85.94 | 139.12±74.51 | 161.0±79.24 | 144.97±113.09 | 0.485 | 0.617 |  |
|  | Men | 163.91±104.88 | 192.38±117.28 | 147.51±101.72 | 152.1±79.23 | 1.631 | 0.199 |  |
|  | FitAA*Sex |  |  |  |  | 1.719 | 0.18 | - |
| **HCY (μmol/L)** | **Whole sample** | **1.11±0.47** | **1.18±0.51** | **1.04±0.45** | **1.19±0.47** | 1.694 | 0.187 | Decel>Accel p=1.00  Decel>Normal p=0.449  Normal<Accel p=0.408 |
|  | Women | 1.15±0.48 | 1.28±0.55 | 1.12±0.48 | 1.13±0.43 | 0.485 | 0.617 |  |
|  | Men | 1.04±0.45 | 1.1±0.49 | 0.91±0.35 | 1.28±0.54 | 1.631 | 0.199 |  |
|  | FitAA*Sex |  |  |  |  | 1.717 | 0.18 | - |
| All data are shown as mean ± standard deviation. *Abbreviations: FitAA, Fitness Age Acceleration; RBC, red blood cells; fL, femtolitre; μL, microliters; μm3, cubic micrometer; hb, hemoglobin; g, grams; mg/dl, milligrams/deciliters; ALP, alkaline phosphatase; U, unit; μg/dL, micrograms/deciliter; ng/mL, nanograms/milliliters; PSH, plasma protein-SH ; MDA,* [*malondialdehyde;*](https://www.google.it/url?sa=t&rct=j&q=&esrc=s&source=web&cd=&ved=2ahUKEwjk6t2Om9KBAxUyQ_EDHSnqCroQFnoECA8QAQ&url=https%3A%2F%2Fwww.ncbi.nlm.nih.gov%2Fpmc%2Farticles%2FPMC3312689%2F&usg=AOvVaw22fO7vC7KP-qogNXPgXoSI&opi=89978449) *μmol/g protein, micromol/g of protein; PON, paraoxonase; HCY, homocysteine.* | | | | | | | | |

|  |
| --- |
